# Long Circulating Lectin Conjugated Paclitaxel Loaded Magnetic Nanoparticles: A New Theranostic Avenue for Leukemia Therapy

**DOI:** 10.1371/journal.pone.0026803

**Published:** 2011-11-16

**Authors:** Abhalaxmi Singh, Fahima Dilnawaz, Sanjeeb Kumar Sahoo

**Affiliations:** Laboratory of Nanomedicine, Institute of Life Sciences, Bhubaneswar, Orissa, India; University of Queensland, Australia

## Abstract

Amongst all leukemias, Bcr-Abl positive chronic myelogenous leukemia (CML) confers resistance to native drug due to multi drug resistance and also resistance to p53 and fas ligand pathways. In the present study, we have investigated the efficacy of microtubule stabilizing paclitaxel loaded magnetic nanoparticles (pac-MNPs) to ascertain its cytotoxic effect on Bcr-Abl positive K562 cells. For active targeted therapy, pac-MNPs were functionalized with lectin glycoprotein which resulted in higher cellular uptake and lower IC_50_ value suggesting the efficacy of targeted delivery of paclitaxel. Both pac-MNPs and lectin conjugated pac-MNPs have a prolonged circulation time in serum suggesting increased bioavailability and therapeutics index of paclitaxel *in vivo*. Further, the molecular mechanism pertaining to pac-induced cytotoxicity was analyzed by studying the involvement of different apoptotic pathway proteins by immunoblotting and quantitative PCR. Our study revealed simultaneous activation of JNK pathway leading to Bcr-Abl instability and the extrinsic apoptotic pathway after pac-MNPs treatment in two Bcr-Abl positive cell lines. In addition, the MRI data suggested the potential application of MNPs as imaging agent. Thus our *in vitro* and *in vivo* results strongly suggested the pac-MNPs as a future prospective theranostic tool for leukemia therapy.

## Introduction

Amongst the different types of leukemias, chronic myelogenous leukemia (CML) is a clonal malignancy of the hematopoietic stem cells that arises from a 9; 22 chromosomal translocation which fuses the ABL proto-oncogene to the BCR gene encoding the fusion protein p210^Bcr-Abl^ [1], [2]. The abnormal p210^Bcr-Abl^ constitutively activates tyrosine kinase activity and promotes leukemogenesis by inducing the phosphorylation of multiple downstream protein targets that mediate multiple growth promoting and anti-apoptotic signals. In mammalian cells, the distinct MAPK members are the important signaling molecules in the control of cell proliferation and differentiation. These include extracellular signal-regulated kinases (ERKs), pAkt, stress-activated protein kinases (SAPKs)/c-Jun N-terminal kinases (JNKs) and the p38 MAPK. The ERK cascade is mainly activated by growth factors and is critical for proliferation and survival. Whereas, JNK and p38 are only weakly activated by growth factors, but are highly activated in response to a variety of stress signals including tumor necrosis factor and their activation is most frequently associated with induction of apoptosis [3]. In CML the Bcr-Abl gene enhances the cell proliferation by activating the ERK and pAkt pathways and by inhibiting the p38 and JNK pathways [1].

Chemotherapy is the main strategy to treat leukemia because unlike solid tumor, hematological malignant tumor cannot be cured by surgical treatment or radiation therapy. However, the major obstacle associated in chemotherapy is the resistance of leukemic cells to various chemotherapeutic agents. This is because most of the anti-cancer drugs induce apoptosis by initiating the intrinsic mitochondrial, or cytochrome *c*/Apaf-1/caspase-9 pathway. The apoptotic pathways in chronic myeloid leukemia are mainly blocked due to Bcr-Abl gene expression, p53 mutation and deficiency of Fas receptor and functional Apaf-1 [4]. To reverse the resistance mechanism with simultaneously reducing the side effects during high dose chemotherapy, a promising approach is rendered through which the conventional chemotherapy could be combined with new strategies leading for the induction of apoptosis to leukemia cells.

Paclitaxel is a potent antineoplastic agent against a wide variety of malignancies. It has been approved by the FDA for the treatment of breast cancer, ovarian cancer and non-small-cell lung cancer (NSCLC) [5]. At the cellular level, Paclitaxel mainly binds to the beta-tubulin subunits in microtubules and promotes polymerization of tubulin and disrupts microtubule dynamics, leading to a sustained mitotic arrest and ultimately to apoptotic cell death [6]. However, molecular pathways involved in the apoptotic process induced by this agent are still not known. Antimicrotubule agents are known to induce phosphorylation and thus inactivation of the antiapoptotic members of the Bcl family such as Bcl-2 [7], [8]. The efficacy of taxanes on human leukemic cell lines [9], [10] as well as their effectiveness in inducing apoptosis *in vivo* [11] and in fresh leukemia cells in primary cultures [12] have been investigated. Al Alami et al. have demonstrated the dose-dependency and time-dependency of the anti-tumor effects of paclitaxel in leukemia [9]. Anti-leukemic activity of taxanes was studied in samples with chromosomal abnormalities associated with an unfavorable outcome, such as the Bcr-Abl translocation (Philadelphia chromosome) [13].

The key problem associated with hematological malignancy is reduced sensitivity of tumor cells to cytotoxic drugs and the drug efflux pumps that gives rise to multi drug resistance (MDR). Nanomaterials are well known to have potential applications in disease diagnosis and therapeutics [14]. The application of the magnetic nanoparticles (MNPs) in the field of biomedical application such as magnetic drug delivery, magnetic resonance imaging, transfection, cell and tissue targeting, has pooled considerable attention due to their intrinsic magnetic properties [15], [16], [17]. The MNPs mediated chemotherapeutics have revealed significant synergistic effect on the apoptosis of leukemic cells [18], [19], [20]. Chen et al. have studied the synergistic effect of gambosic acid and daunomycin on the drug accumulation and apoptosis of leukemia cells intervened by iron oxide (Fe_3_O_4_) nanoparticles [21]. In another study, Lv et al. have demonstrated the efficacy of the Fe_3_O_4_-polylactic acid (PLA) nanocomposites for increased local drug concentration at the drug-sensitive leukemia K562 cells leading to induction of cell death [22].

To utilize the MNPs as a drug delivery vehicle, it is essential to be coated with hydrophilic or hydrophobic polymer to avoid the aggregation *in vivo*. Further, these MNPs should also acquire high drug loading capacity, desired release profile, aqueous dispersibility, biocompatibility with cells and tissues along with retention of magnetic properties [23]. Our previous study have demonstrated that coating the MNPs with long chain amphiphilic lipid polymer glyceryl monooleate (GMO) provides the aqueous dispersibility and permits the incorporation of both hydrophilic and hydrophobic drugs [15], [24]. In different experimental setups, our group has already established the fact that, targeted drug delivery through functionalized drug carriers is an effective strategy to provide higher therapeutic concentrations of anticancer drugs by overcoming the multidrug resistance at the targeted cancer cells thereby reducing the dose and unaffecting the noncancerous tissues [25], [26], [27]. Apart from the therapeutic application, currently significant attention has been laid down for the multifunctional characteristics and complementary role of MNPs as a contrast agent for the magnetic resonance imaging (MRI) [24], [28], [29]. MRI provides excellent differential soft tissue contrast that helps to discriminate healthy tissues and abnormal tissues like cancer tissues. Thus, magnetic nanoparticles provide an excellent diagnosis tool for the treatment of cancer. In addition, in case of leukemia, T_2_ weighted MRI has already been established as an efficient diagnostic and management tool [30], [31]. Jaetao et al. have demonstrated that detection of leukemic cells can be enhanced by using MNPs with surface conjugation of receptor-specific ligands [32].

So, here in this study, we have investigated the efficacy of paclitaxel loaded MNPs (pac-MNPs) formulations functionalized with a targeting moiety lectin. The use of specific molecular signatures at the targeted site helps in delivering the appropriate therapeutic concentration aiding the advantage of receptor mediated endocytosis. The cellular uptake efficiency of pac-MNPs and lectin conjugated pac-MNPs (lec-pac-MNPs) was compared with native drug in leukemic cell line (K562). The uptake experiment was also studied in lectin negative cell line i.e, HEK293. To elucidae the probable molecular pathways ascribed through pac-MNPs in K562 cells, the levels of different extrinsic and intrinsic apoptotic pathway proteins were studied through immunobloting. Besides the levels of expression of different apoptotic genes and CML specific Bcr-Abl gene were quantified through real time PCR following post treatment of native pac, pac-MNPs and lec-pac-MNPs. To validate the outcome, the gene expression of apoptotic pathway proteins and Bcr-Abl were studied in another Bcr-Abl positive cell line i.e, Kcl22 and Bcr-Abl negative cell line i.e, Jurkat.

## Results

### Physicochemical characterization of Pac-MNPs

**Figure 1:**
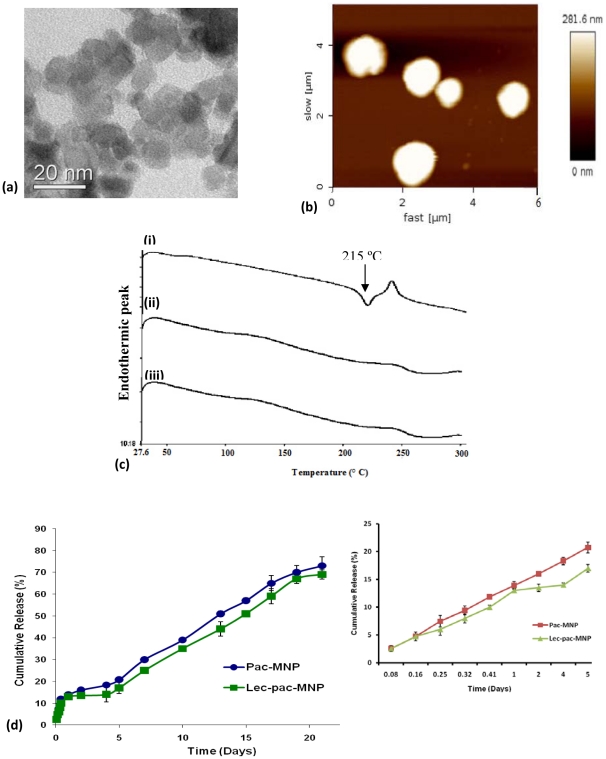
Physicochemical characterization of pac-MNPs. (A) TEM figure of pac-MNPs. (B) AFM figure of pac-MNPs. (C) DSC curves of (i) native pac, (ii) void MNPs and (iii) pac-MNPs. (D) Release kinetics of paclitaxel from pac-MNPs and lec-pac-MNPs (data as mean ± SEM, n = 3). The insert represents the release of paclitaxel from pac-MNPs and lec-pac-MNPs up to 5^th^ day (data as mean ± SD, n = 3).

The synthesis and the physicochemical characterizations of our formulated magnetic nanoparticles were well studied and already described and reported by our group [15]. The internal diameter of the MNPs measured by TEM was ∼7 nm (Figure 1 A). However, the pac-MNPs illustrated hydrodynamic diameter ∼200 nm as measured by the Zetasizer (Zetasizer Nano ZS, Malvern Instruments, Malvern, UK). The AFM microscopy revealed the spherical topology of the nanoparticles (Figure 1 B). The AFM observation also substantiates the hydrodynamic size of the particles. The difference in hydrodynamic and TEM size is may be due to the fact that it provides information of the hydrodynamic layers formed due to the coatings around the cluster of pac-MNPs [15]. The pac-MNPs have high negative zeta potential ∼−23 mV as measured by dynamic laser light scattering [15].

Differential scanning calorimetry (DSC) provides the information about possible chemical interaction and the physical state of drug inside the nanoparticles [33]. As per the DSC results, the native pac showed an endothermic peak at 215°C (Figure 1C-i). At this range, no peak was observed in case of void MNPs (Figure 1C-ii), moreover there was absence of endothermic peak for pac in pac-MNPs (Figure 1C-iii). The absence of specific peaks for the crystalline domain of native pac suggested that the drug present inside the MNPs formulation is in amorphous or disordered-crystaline state [34]. The *in vitro* release kinetics profile showed release of 73±4% pac from pac-MNPs and 69±2.1% from the lec-pac-MNPs for a period of 20 days (Figure 1 D). The results showed initial burst release followed by slow and sustained release [15].

### *In vitro* Imaging

**Figure 2:**
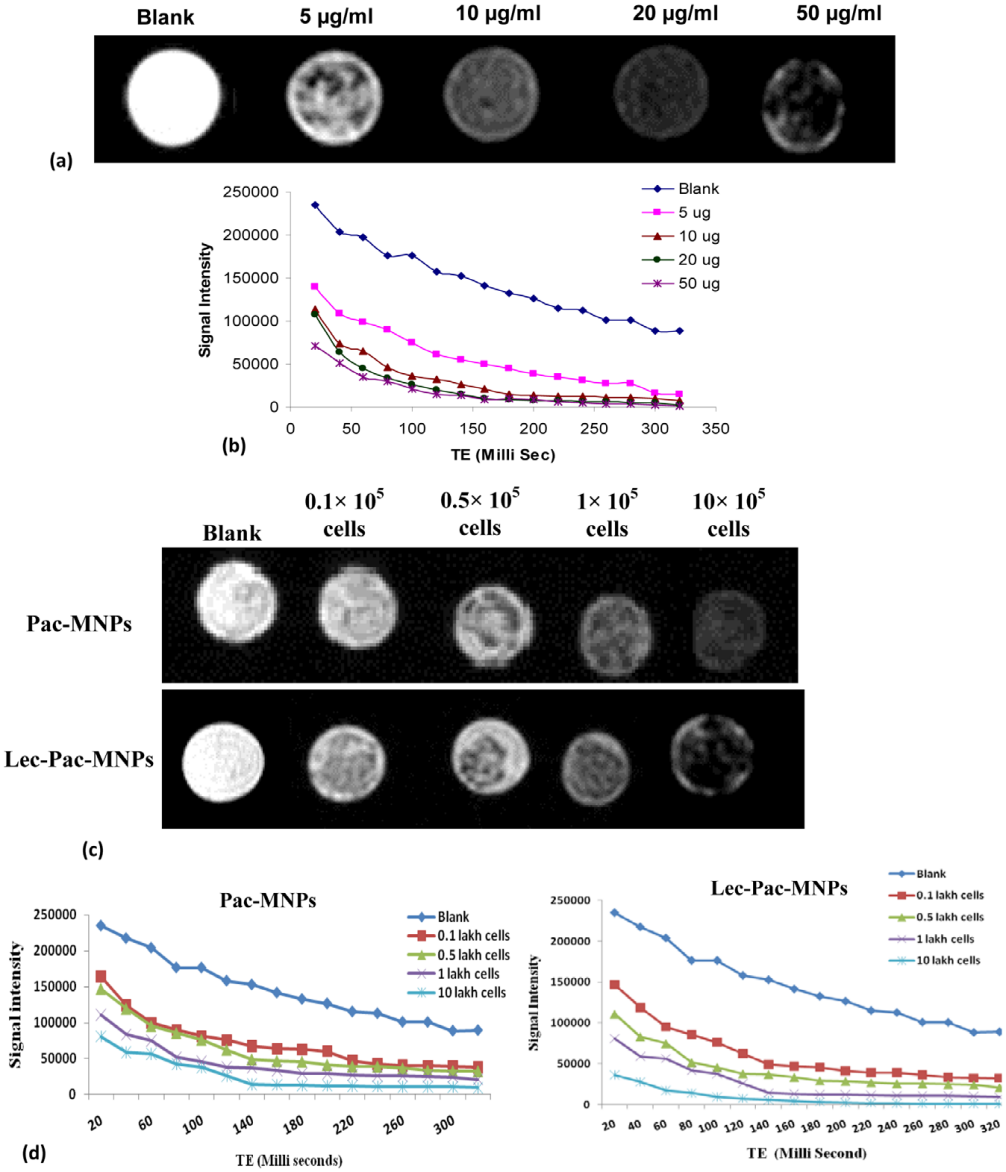
MRI study. (A) MR signal intensity of pac-MNPs in phantom agar gel at various iron concentration, blank phantom agar gels were taken as control. (B) T_2_ relaxation analysis curves of Pac-MNPs in phantom agar gel at different concentration (data as mean intensity within region of interest (ROI)). (C) MR signal intensity of pac-MNPs and lec-pac-MNPs treated K562 cells in phantom agar gel at various cell concentration, blank phantom agar gels were taken as control. (D) T_2_ relaxation analysis curves of Pac-MNPs and lec-pac-MNPs treated K562 cells in phantom agar gel at different cell concentration (data as mean intensity within region of interest (ROI)).

To evaluate the usefulness of the drug loaded MNPs as a contrast agent, the imaging characteristics of pac-MNPs were evaluated in a phantom gel. The transverse relaxation time T_2_ was observed to be reduced as the concentration of the pac-MNPs (measured in µg Fe/ml) were increased in the phantom gels compared to the control gel (Figure 2 A). In addition, with increase in particle concentration of MNPs the reduction of T_2_ was more significant; this was evident from the decreased signal intensity (Figure 2 B). This reduction in T_2_ relaxation value suggests the pac-MNPs to be a good candidate for imaging in MRI [24]. In addition, to prove whether after uptake of the MNPs the leukemia cells can be detected by the MRI, T_2_ relaxation of pac-MNPs and lec-pac-MNPs treated K562 cells was measured. The T_2_ relaxation was observed to be reduced as the cell number was increased from 0.1 lakh to 10 lakh (Figure 2 C). The reduction of T_2_ relaxation can be observed from the signal intensity curve of the different concentration of pac-MNPs and lec-pac-MNPs treated K562 cell number (Figure 2 D). While comparing the pac-MNPs and lec-pac-MNPs treated K562 cells it can be observed that in case of lec-pac-MNPs treated K562 cells, there is higher reduction in the signal intensity (Figure 2 D).

### Uptake enhancement through lectin conjugation

**Figure 3:**
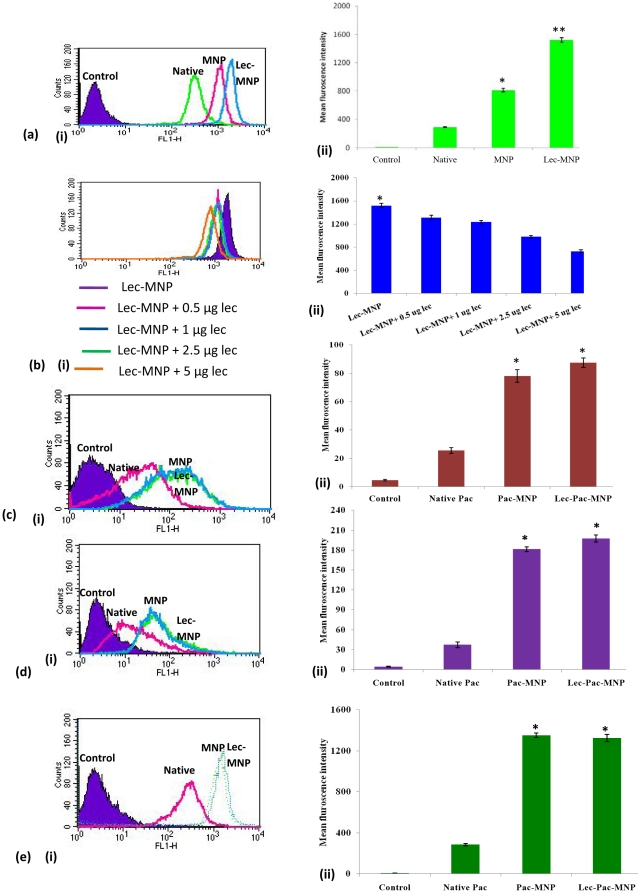
Uptake studies. (A) Uptake analysis of native 6-coumarin, 6-coumarin-MNPs and lec-6-coumarin-MNPs in K562 cells (i) through flow cytometry (ii) Mean fluorescence intensity obtained from flow cytometry. (B) Competitive inhibition assay study for uptake of lec-6-coumarin-MNPs by adding different concentrations of native lectin in K562 cells (i) through flow cytometry (ii) Mean fluorescence intensity obtained from flow cytometry. (C) Uptake analysis of native 6-coumarin, 6-coumarin-MNPs and lec-6-coumarin-MNPs in Kcl22 cells (i) through flow cytometry (ii) Mean fluorescence intensity obtained from flow cytometry. (D) Uptake analysis of native 6-coumarin, 6-coumarin-MNPs and lec-6-coumarin-MNPs in Jurkat cells (i) through flow cytometry (ii) Mean fluorescence intensity obtained from flow cytometry. (E) Uptake analysis of native 6-coumarin, 6-coumarin-MNPs and lec-6-coumarin-MNPs in HEK293 cells (i) through flow cytometry (ii) Mean fluorescence intensity obtained from flow cytometry. Data as mean ± S.E.M., n = 6. (*) p<0.05, (**) p <0.01.

Human C-type lectin like molecules are the transmembrane glycoproteins expressed in myeloid cells derived from peripheral blood and bone marrow [35], [36]. To compare the uptake of native drug, MNPs and lectin conjugated MNPs, 6-coumarin was taken as a probe and FACS analysis was performed. The mean fluorescence intensity results showed that the cellular uptake of lec-6-coumarin-MNPs was ∼4 times higher than the native 6-coumarin and ∼1.8 times higher than 6-coumarin -MNPs (Figure 3 A). To supplement the fact that uptake of lectin conjugated MNPs is through receptor mediated endocytosis, we have done a competitive inhibition assay. The results showed that addition of 0.5 µg native lectin to the above formulation reduced the uptake of lec-6-coumarin-MNPs by ∼1.5 fold and a gradual decrease was observed with increasing concentration of lectin (Figure 3 B). Further, we have checked the uptake efficiency in another Bcr-Abl positive cell line Kcl22 (Figure 3 C) and a Bcr-Abl negative cell line Jurkat (Figure 3 D) and both of them do not show overexpression of lectin receptors.

In both the cell lines, there was a significant higher uptake of 6-coumarin-MNPs than the native one (∼4 times in case of Kcl22 and ∼5 times in case of Jurkat) whereas, the difference in the uptake of 6-couma-MNPs and lec-6-coumarin-MNPs was minimal. The enhanced uptake due to the lectin receptor was further authenticated taking a lectin negative cell line i.e, HEK293. The uptake analysis revealed that the MNPs showed an enhancement in uptake by ∼5 times but the uptake in case of lec-MNPs was similar to that of MNPs.

### *In vivo* Pharmacokinetics

**Figure 4:**
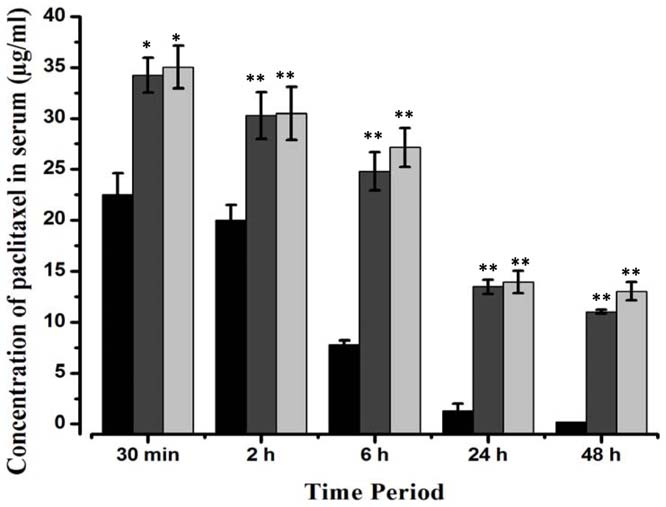
*In vivo* bioavailability of native pac (▪), pac-MNPs (▪) and lec-pac-MNPs (▪) in a rat model. The rats were divided in three groups. Equivalent concentration of pac, pac-MNPs and lec-pac-MNPs (20 mg/kg) was administered intravenously. Blood was collected at different time intervals and paclitaxel concentration in serum was determined by RP-HPLC analysis, as described in materials and methods. (**) p<0.01 and (*) p<0.05 pac versus pac-MNPs.

*In vivo* pharmacokinetics was performed to compare the bioavailability of paclitaxel in the blood serum of rats after intravenous injection of native pac, pac-MNPs and lec-pac-MNPs at different time periods up to 48 h. Figure 4 shows that the highest concentration of paclitaxel in case of native pac, pac-MNPs as well as lec-pac-MNPs can be observed after 30 minutes of injection. After 24 h of treatment, the difference in the paclitaxel concentration in serum between native pac and both the formulations is very high. In addition, after 48 h, in case of native pac treated rats, the blood serum shows un-detectable concentration of paclitaxel whereas, in case of pac-MNPs and lec-pac-MNPs treated rats, the concentration of paclitaxel in serum was 11 µg/ml and 13 µg/ml respectively. The pharmacokinetics analysis suggests that, in case of native pac, the T_1/2_ is achieved at only 5 h. (Table 1). But, in case of pac-MNPs and lec-pac-MNPs the T_1/2_ is reached after 15 h. The C_max_ for native pac is 22.4 µg/ml whereas for pac-MNPs and lec-pac-MNPs it is 34.2 µg/ml and 35 µg/ml respectively (Table 1). These data suggests the longer blood circulation half-life of pac-MNPs and lec-pac-MNPs.

**Table 1:** Pharmacokinetics of native pac, pac-MNPs and lec-pac-MNPs in rat model.

Group of rat treated with	C_max_ (µg/ml) ± S.D.	T_1/2_ (h) ± S.D.
Native pac	22.4±2.1	5.2±0.08
Pac-MNPs	34.2±1.7	15.2±0.09
Lec-pac-MNPs	35.0±2.5	15.7±0.13

### Cytotoxicity effect of different formulation on K562 cells

**Figure 5:**
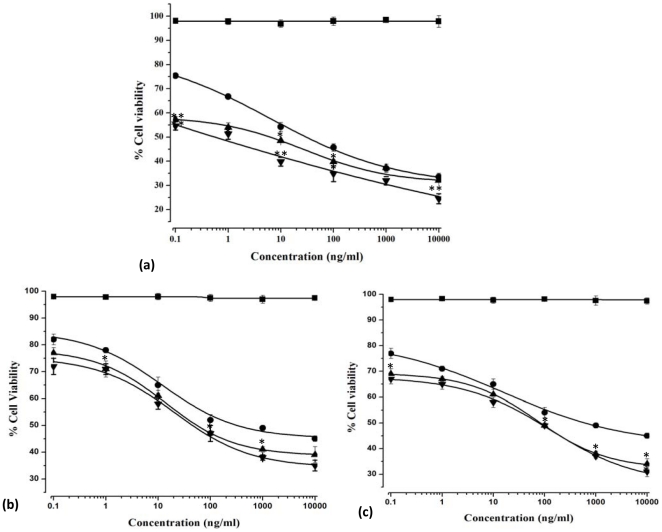
Dose dependent cytotoxicity studies of Void MNP (▪), native pac (•), pac-MNPs (▴) and lec-pac MNPs (∇) in (A) K562 cells, (B) Kcl22 cells and (C) Jurkat cells after 48 h. The extent of growth inhibition was measured by the MTT assay. The inhibition was calculated with respect to control (cells treated with only medium). Data as mean ± S.E.M., n = 6. (*) p<0.05, drug in solution versus drug in MNPs, (**) p<0.01 drug in solution versus lec-pac-MNPs.

We have tested the cytotoxicity of void MNPs and confirmed these to be nontoxic [15]. The therapeutic efficacies of paclitaxel at concentrations of different native pac, pac-MNPs and lec-pac-MNPs in K562, Kcl22 and Jurkat cells were investigated by mitogenic assay. The results showed a typical dose dependent sigmoidal antiproliferative effect of drug and drug loaded different formulations. The half maximal inhibitory concentration (IC_50_) values, calculated from the obtained sigmoidal curves, demonstrated that the lec-pac-MNPs showed higher antiproliferative activity than un-conjugated pac-MNPs and the native pac in K562 cells (Figure 5 A).

After 48 h of treatment, the lec-pac-MNPs are ∼67 times more effective than native pac and ∼10 times more effective than the pac-MNPs in K562 cell line (Table 2). Similarly, in case of another Bcr-Abl positive cell line Kcl22 and the Bcr-Abl negative Jurkat cells, native pac, pac-MNPs and lec-pac-MNPs induced cytotoxicity after 48 h of treatment. But, as the above two cell lines do not overexpress the lectin receptors, there was minimal difference between the IC_50_ values of pac-MNPs and lec-pac-MNPs. The pac-MNPs were 5.7 and lec-pac-MNPs were 6.9 times more effective than the native pac in Kcl22 cells (Figure 5 B, Table 2). In Bcr-Abl negative cell line Jurkat also, cytotoxicity effect of native pac, pac-MNPs and lec-pac-MNPs can be observed after 48 h. (pac-MNPs were 7.3 and lec-pac-MNPs were 7.8 times more effective than the native pac) as shown in Figure 5 C, Table 2.

**Table 2:** IC_50_ values of native pac, pac-MNPs and lec-pac-MNPs in three leukemia cell lines.

Sample	IC_50_ (ng/ml) ± S.D.			
	K562	Kcl22	Jurkat	
Native pac	40.6±1.4	319±9.2		609±11.7
Pac-MNPs	6.3±0.7	55.2±2.2		83.1±7.4
Lec-pac-MNPs	0.6±0.02	45.9±3.1		77.2±6.9

### Lectin conjugation enhances G_2_-M Arrest

**Figure 6:**
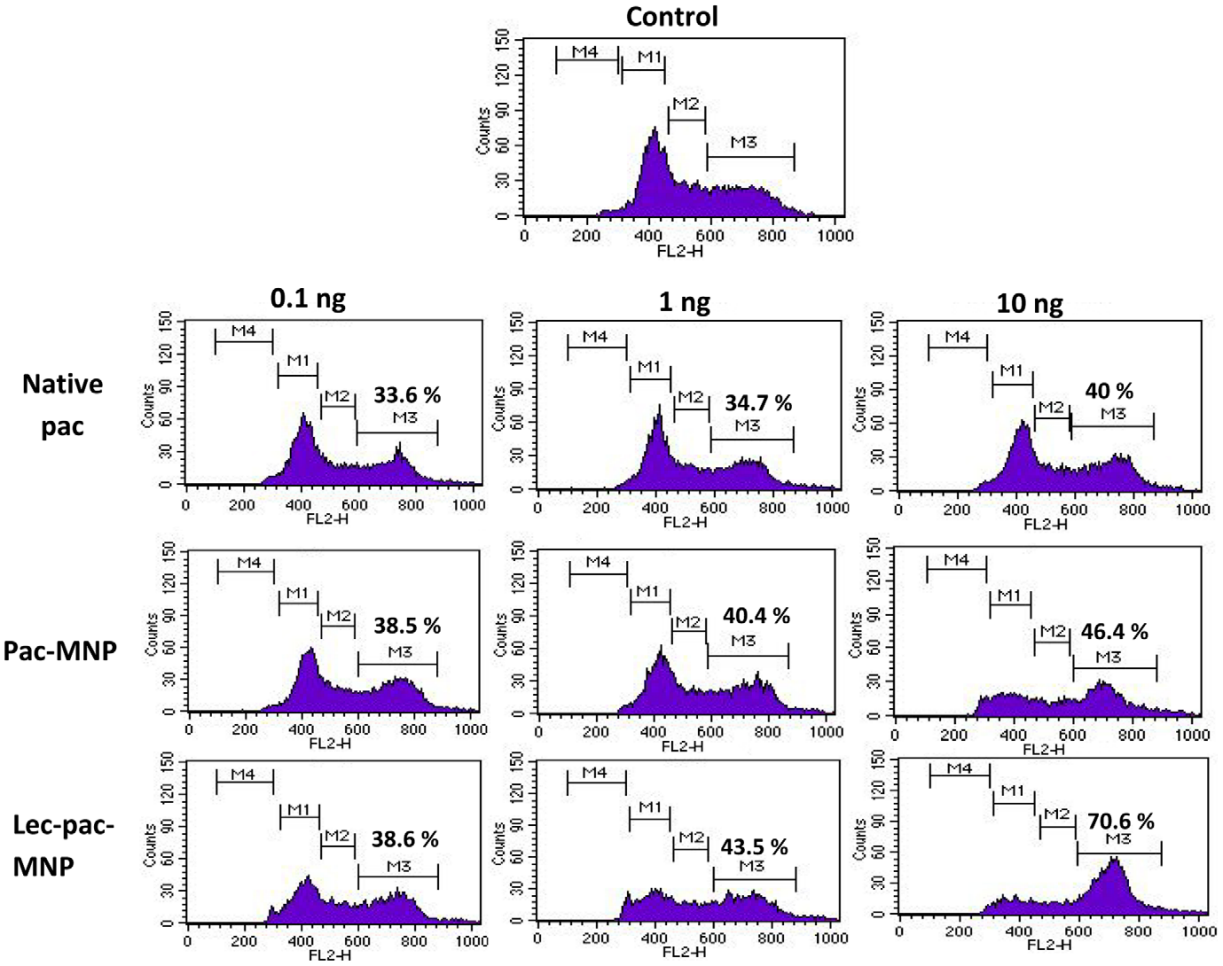
Cell cycle analysis of K562 cells after 24 h of treatment with native pac, pac-MNPs and lec-pac-MNPs at concentration of 0.1 ng, 1 ng and 10 ng/ml. The figure shows the percentage of cells at G_2_-M phase of cell cycle.

Paclitaxel induces G_2_-M arrest due to its stabilizing effects on microtubules. FACS analysis demonstrated that paclitaxel strongly arrest K562 cells in G_2_-M phase (Figure 6) and a greater proportion of cells in G_2_-M phase was observed in cells treated with lec-pac-MNPs as compared to pac-MNPs. At the concentration 0.1 ng/ml, native pac showed only 33.6% in G_2_-M phase but, both the nano formulations (unconjugated and conjugated) showed ∼38% cells in G_2_-M phase arrest. As evident from the figure, with increasing concentration of drug, the arrest of cells at G_2_-M phase increased. At the concentration 10 ng/ml the native pac and pac-MNPs showed 40% and 46% cells in G_2_-M phase arrest, whereas, lec-pac-MNPs showed the highest 70.6% cells in G_2_-M phase arrest. As compared to other formulations, lec-pac-MNPs at concentration of 10 ng/ml exhibited significant G_2_-M phase arrest.

### Lectin conjugated pac-MNPs induces more apoptosis

**Figure 7:**
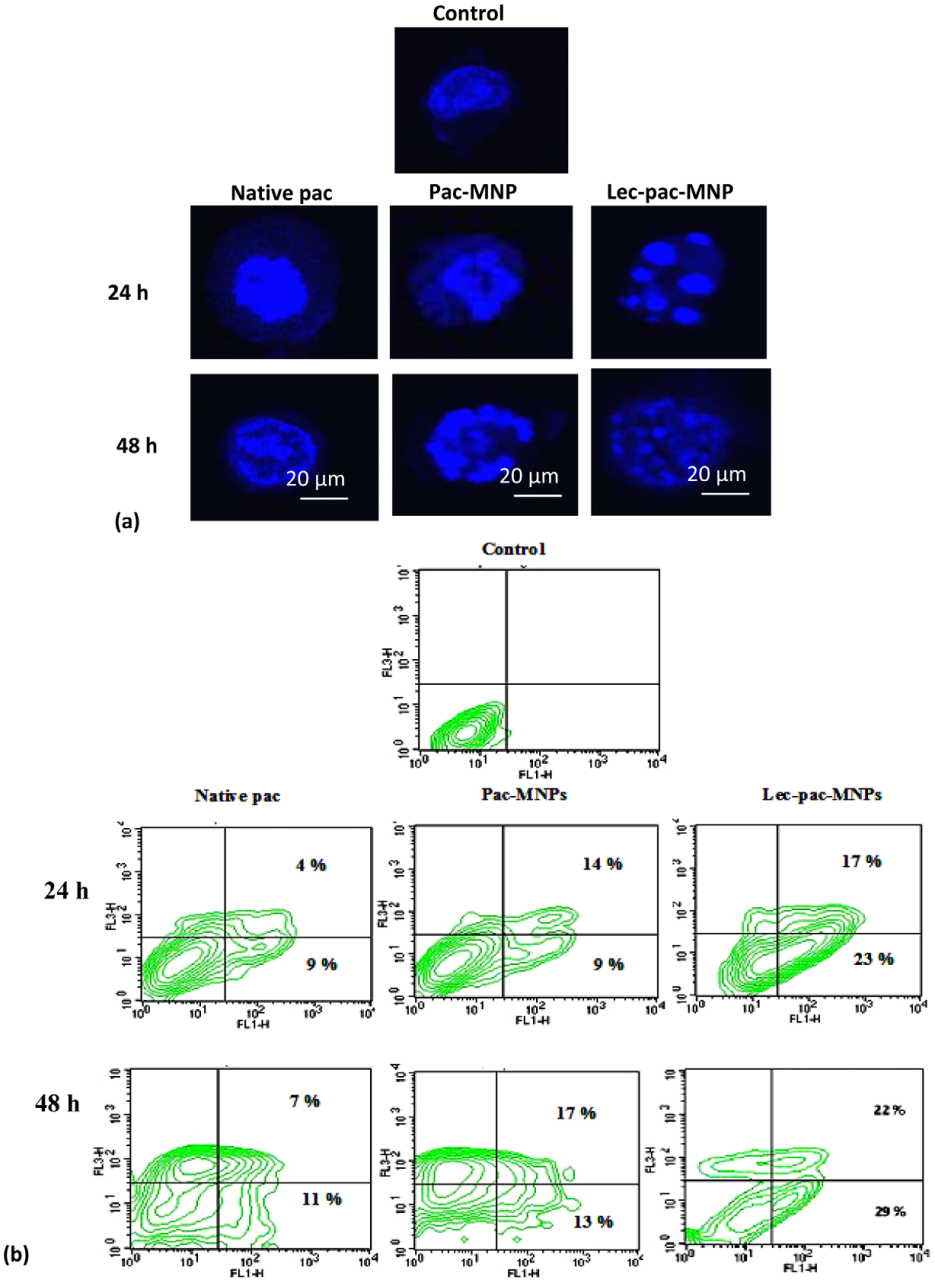
Apoptosis study in K562 cells. (A) Morphological analysis of K562 cells after treatment with native pac, pac-MNPs and lec-pac-MNPs (Conc. 10 ng/ml) after 24 and 48 h by confocal microscopy. (B) Induction of apoptosis in K562 cell line by native pac, pac-MNPs and lec-pac-MNPs (Conc. 10 ng/ml) after 24 and 48 h using Annexin V- FITC. Flow cytometry analysis reveals the presence of different populations of cells. Top right: late apoptotic and necrotic cells; bottom left: live cells and bottom right: early apoptotic cells.

The induction of apoptosis in K562 cells by native pac, pac-MNPs and lec-pac-MNPs was studied by structural analysis through confocal microscopy and quantitatively through FACS analysis. The cellular structural integrity study by confocal microscopy revealed gradual nuclear condensation in early stages which later on led to nuclear fragmentation (Figure 7 A). In case of the targeted nanoparticles, maximum nuclear fragmentation was observed even at 24 h of treatment as the blebbing of the nucleus was clearly visible (Figure 7 A). The use of Annexin V FITC and propidium iodide staining used for differentiation of apoptotic cells in different stages suggest that after treating K562 cells with 10 ng/ml of drug, in 24 h, the lec-pac-MNPs showed ∼40% apoptotic population which increased to ∼51% in 48 h whereas the pac-MNPs and native pac showed ∼22% and ∼13% respectively which increased to 30% and ∼18% after 48 h (Figure 7 B). After confirming the lec-pac-MNPs to be the better formulation to induce apoptosis in K562 cells, we studied the detailed molecular mechanism through which the drug induced apoptosis by immunoblotting and real time PCR.

### Immunoblotting experiment showing activation different molecular pathways

**Figure 8:**
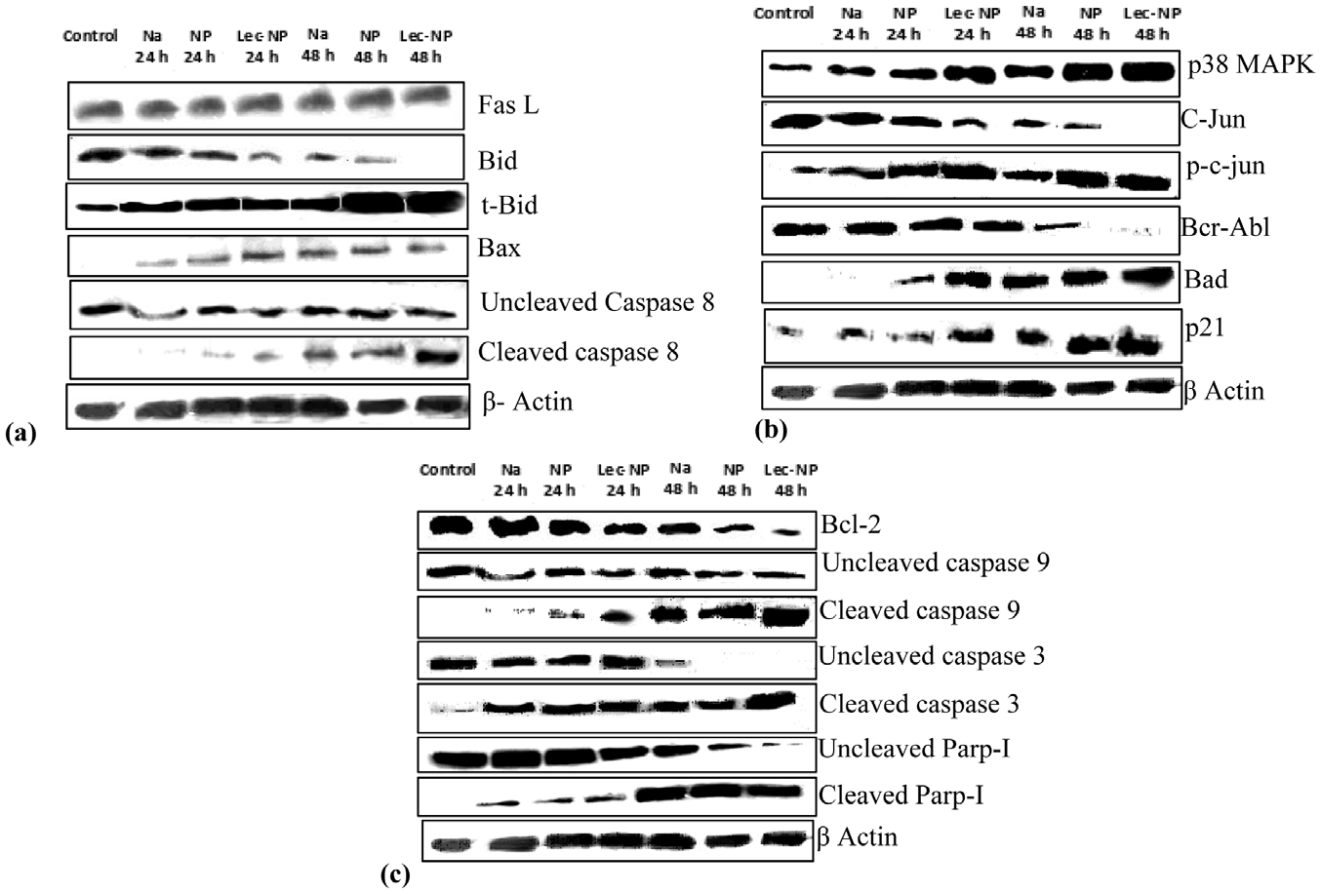
Immuno-blot analysis of proteins apoptotic pathway. (A) extrinsic pathway (B) JNK pathway and (C) intrinsic pathway proteins in K562 cells treated with 10 ng/ml of native pac, pac-MNPs and lec-pac-MNPs (details described in materials and methods.

K562 cells have been reported to resist the TNF-α induced extrinsic apoptotic pathway [4]. Pac formulations are not sensitive towards Fas-L mediated cell death as the level of Fas-L in treated cells was similar to that of untreated cells even after 48 h of treatment (Figure 8 A). However, pac-MNPs induced time dependent cleavage of extrinsic pathway protein caspase 8 and the cleavage was more significant in case of lec-pac-MNPs (Figure 8 A). These results provided the proof that activation of extrinsic apoptotic pathway is in a Fas-L independent manner and might be TRAIL (TNF related apoptosis inducing ligand) dependent. Further, caspase 8 induced the cleavage of proapototic signaling protein Bid. A noticeable decrease in the level of full length Bid and increase in the level of truncated Bid (t-Bid) was observed, which was maximum for lec-pac-MNPs after 48 h of treatment (Figure 8 A). The protein t-Bid facilitate the insertion of Bax into mitochondrial membrane and links with the intrinsic apoptotic pathway. The increased level of Bax protein could be observed from the western blotting result (Figure 8 a).

Another crucial factor in K562 cells is Bcr-Abl which is a constitutively active protein tyrosine kinase, promotes growth factor independent cell proliferation by activating PI3K/AKT, Stat 5 and Ras/ MEK/ Erk pathways and upregulating the antiapoptotic proteins [1]. It was observed that after 48 h of treatment with pac formulations, the level of Bcr-Abl protein decreased significantly. The decrease was more significant in case of lec-pac-MNPs as compared to pac-MNPs (Figure 8 B). Further, we have studied the possible mechanism behind down regulation of *Bcr-Abl* gene. Paclitaxel is known to cause apoptosis through activation of stress signaling pathways like p38MAPK and JNK pathways [37]. It was observed that even after 24 h of treatment, the level of p38MAPK increased and the significant higher level was observed after 48 h (Figure 8 B).

Further, activation of JNK pathway became apparent from the results showing increased level of phosphorylated c-Jun and decreased level of c-Jun after drug treatment (Figure 8 B). Activation of JNK can cause instability of Bcr-Abl complex [38]. Induction of stress signaling pathways along with downregulation of Bcr-Abl complex induces programmed cell death through induction of proapototic signal proteins like Bad and p21 [39], [40]. It was apparent from the results that, there was an increased level of Bad and p21 (Figure 8 B) in a p53 independent manner, as p53 is in a mutated state in K562. Also, there was decreased level of antiapoptotic signal proteins like Bcl-2 (Figure 8 C*).* With down regulation of Bcl-2, the proapoptotic proteins Bad and Bax caused permiabilization of the mitochondrial membrane potential leading to release of cytochrome c. With the release of cytochrome c from mitochondria, the caspase cascade gets activated, which was evident from the immunoblot and the quantitative PCR results. The released cytochrome c stimulated cleavage of caspase 9 which was more significant after 48 h of treatment and was maximum in case of the lec-pac-MNPs (Figure 8 C). The caspase 9 from intrinsic pathway and the caspase 8 from extrinsic pathway directed the cleavage of caspase 3 and finally lead to apoptosis. This was evident from the decreased level of uncleaved caspase 3 and increased level of cleaved caspase 3 (Figure 8 C).

### Mitochondrial membrane depolarization confirming initiation of intrinsic pathway of apoptosis

**Figure 9:**
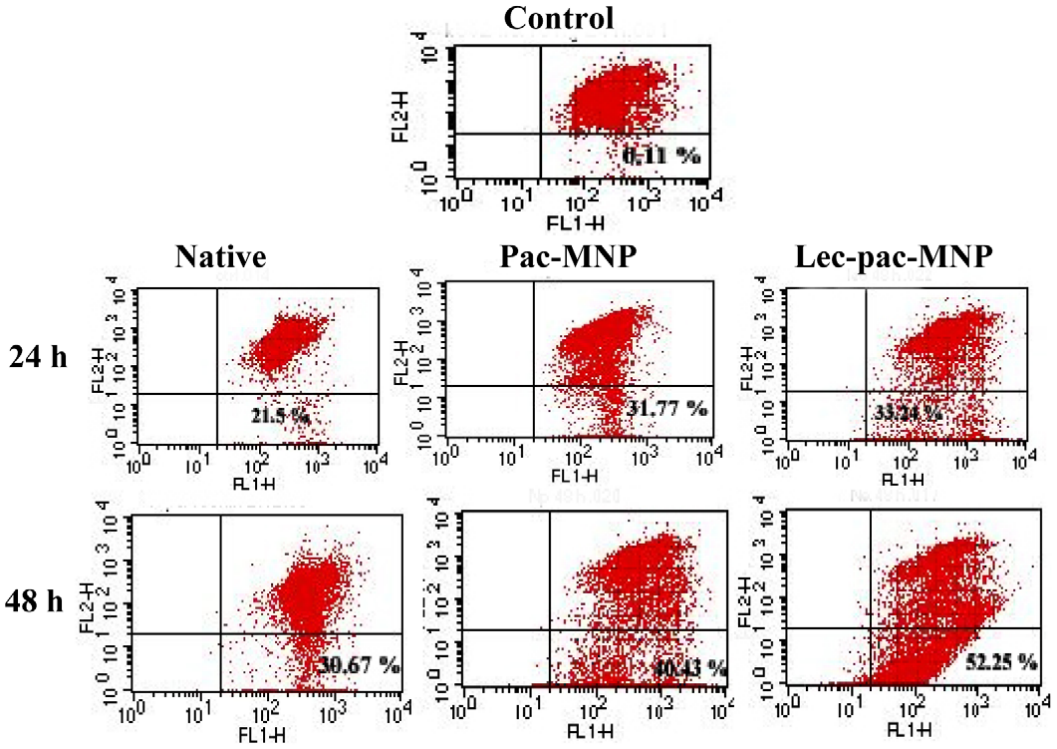
Changes in mitochondrial membrane potential measured through JC-1 dye after 24 and 48 h of treatment with native pac, pac-MNPs and lec-pac-MNPs (10 ng/ml) in K562 cells.

Loss of mitochondrial membrane potential is one of the key features revealing the initiation of intrinsic apoptotic pathway which can be studied using JC-1 dye [41]. The FACS analysis in treated K562 cells suggested a time dependent loss of membrane potential which was highest in case of lec-pac-MNPs that might be due to the higher uptake of lec-pac-MNPs. After 24 h of treatment, the native pac and pac- MNPs showed only ∼21.5 and 31.77% apoptotic population which increased to 30.67 and 40.43% respectively after 48 h of treatment (Figure 9). Similarly, the lec-pac-MNPs showed only ∼33.24% apoptotic population after 24 h of treatment which increased to 52.25% after 48 h (Figure 9).

### Quantitative gene expression study of apoptotic pathway by real time PCR

**Figure 10:**
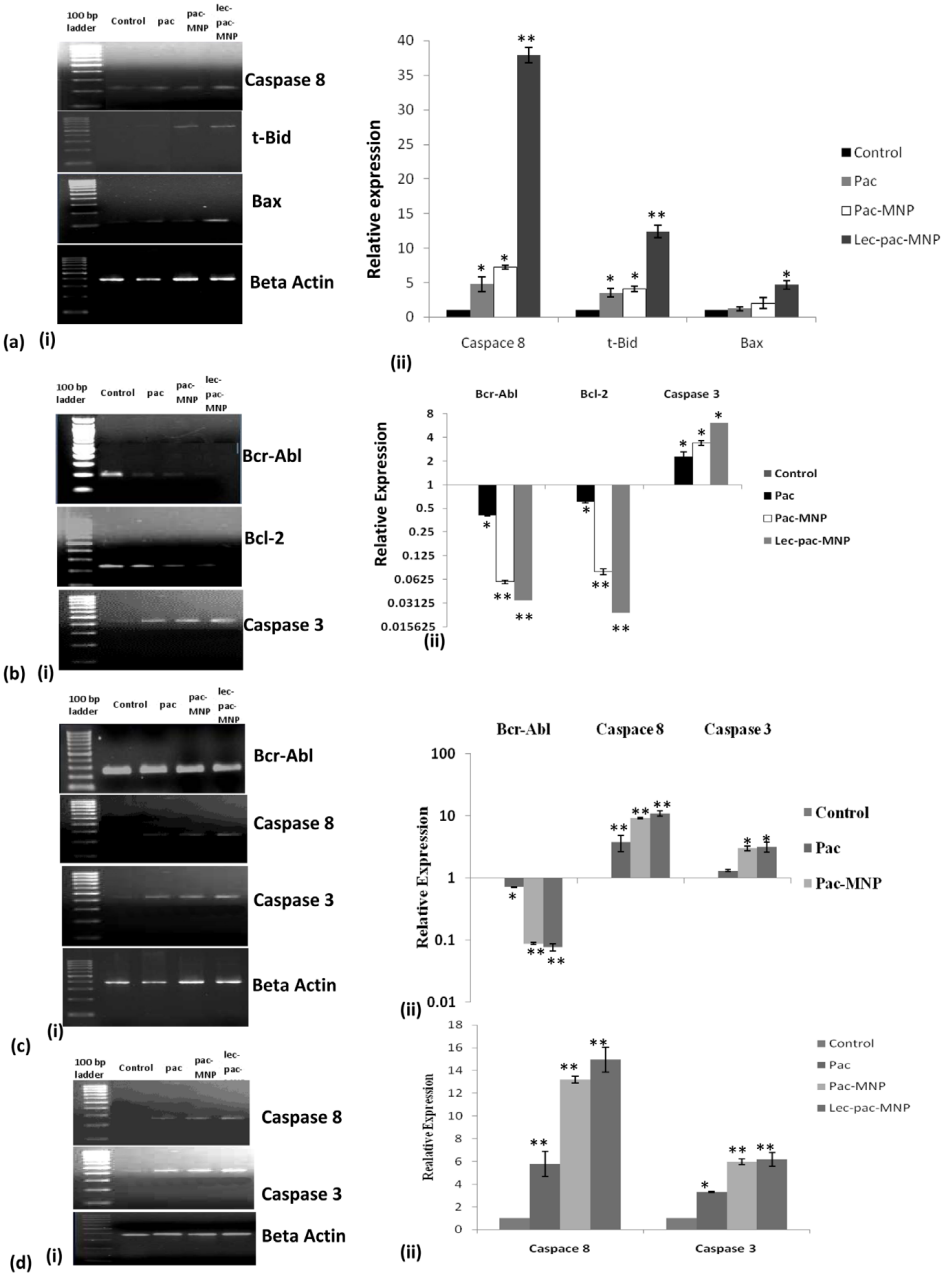
Gene expression study by real time PCR. (A) (i) m-RNA expression (ii) quantification of Caspase 8, Bid, and Bax gene by RT-PCR in K562 cell lines. (B) (i) m-RNA expression (ii) quantification of Bcr-Abl, Bcl-2 and caspase 3 gene by RT-PCR in K562 cell lines. (C) (i) m-RNA expression (ii) quantification of Bcr-Abl, Caspase 8 and caspase 3 gene by RT-PCR in Kcl22 cell lines. (D) (i) m-RNA expression (ii) quantification of Caspase 8 and caspase 3 gene by RT-PCR in Jurkat cell lines. (**) p<0.01 and (*) p<0.05 control versus pac, pac-MNPs and lec-pac-MNPs. (Details described in materials and methods).

To study the expression of the apoptotic pathway factors at the mRNA level and to deduce the fold increase or decrease in the expression, quantitative PCR was done. For the extrinsic pathway factors, in case of caspase 8, the lec-pac-MNPs showed 38 fold increase in the expression of cleaved caspase 8 whereas the the pac-MNPs showed 7 fold and the native pac showed only 5 fold increase in the expression (Figure 10 A i, ii). Similarly, the lec-pac-MNPs showed 12 fold increase in the expression of t-Bid whereas native pac and pac-MNPs showed 3 and 4 fold increase respectively than control t-Bid (Figure 10 A i, ii). Likewise, in case of Bax, the lec-pac-MNPs showed 5 fold higher increase in the expression (Figure 10 A i, ii). For the CML specific Bcr-Abl gene, the quantitative PCR results showed that the fold decrease in the expression in case of lec-pac-MNPs and pac-MNPs treated cells were 29 and 17 fold respectively whereas that of native pac was 2.4 fold compared to untreated cells (Figure 10 B i, ii). Also, there was decreased level of antiapoptotic signal proteins like Bcl-2 (Figure 10 B i, ii) and the decrease was most significant in case of lec-pac-MNPs i.e, 42.4 fold (Figure 10 B i, ii). Finally, the quantitative PCR, revealed 16 fold increase in the level of cleaved caspase 3 in case of lec-pac-MNPs and 8 and 1.3 folds increase respectively in case of pac-MNPs and native pac as compared to control cells (Figure 10 B i, ii).

To supplement the molecular pathway of apoptosis revealed in K562 cells, gene expression of some key factors were studied in another Bcr-Abl positive cell line Kcl22 and Bcr-Abl negative Jurkat cells. In Kcl22, after 48 h of treatment, the down regulation of Bcr-Abl gene was observed (Figure 10 C i, ii). The fold decrease in the expression of Bcr-Abl gene was 1.4 fold in case of native Pac and 12.5 fold in case of pac-MNP. For the extrinsic apoptotic pathway factor, caspase 8, the increase in the expression was observed in both Kcl22 and Jurkat cell lines. The native Pac showed 3.7 fold and 5.7 fold increase in the level of cleaved caspase 8 in case of Kcl22 and Jurkat respectively whereas the Pac-MNPs sowed 9.1 fold and 13.1 fold increase respectively as compared to the control cells (Figure 10 C, D). Finally, increase in the expression of apoptotic factor caspase 3 was observed in both Kcl22 and Jurkat cell line. The native Pac showed 1.3 fold and 3.3 fold increase in the level of cleaved caspase 8 in case of Kcl22 and Jurkat respectively whereas the Pac-MNPs sowed 2.9 fold and 5.9 fold increase respectively as compared to the untreated control cells (Figure 10 C, D). In both Kcl22 and Jurkat cells, the level of expression of different genes in case of pac-MNPs treatment and lec-pac-MNPs treatment was similar.

**Figure 11:**
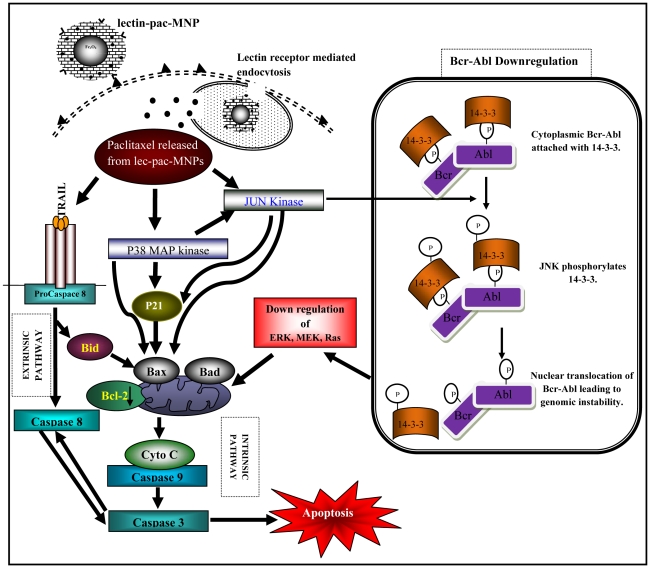
Schematic diagram showing the receptor mediated endocytosis of lec-pac-MNPs and mechanism of action of paclitaxel after released from the nanoformulation showing coordinated regulation of signaling cascades leading to apoptosis.

The above results from western blotting and real time PCR suggest that the lec-pac-MNPs after entering into the leukemic cells by receptor mediated endocytosis, can exert apoptotic effect by both extrinsic and intrinsic apoptotic pathways. The extrinsic pathway induces cleavage of caspase 8 which is further linked to the either mitochondrial pathway or directly to the caspase 3 cleavage (Figure 11). The intrinsic pathway may regulate the MAPKinase pathway which further joins the mitochondrial paoptotic pathway by p21. In case of leukemia, paclitaxel may help in Bcr-Abl gene instability by enhancing the Jun Kinase which may have led to 14-3-3 protein phosphorylation which remains linked to Bcr-Abl (Figure 11).

## Discussion

At present most of the therapeutic modes for CML, includes chemotherapy, interferon mediated immunotherapy and bone marrow transplantation. The primary cause of treatment failures in leukemia is due to the resistance of leukemic cells to chemotherapy-induced apoptosis and emergence of MDR. The *Bcr-Abl* gene in CML activates the signaling pathways like PI3K/AKT, Ras etc that confer growth factor independent proliferation [1]. In addition, CML cells are resistant to induction of apoptosis by variety of agents, such as TNFα, CD95/FasL etc. [4]. Although, the advent of the abl tyrosine kinase inhibitor, imatinib, has revolutionized the treatment of CML [42], approximately 30% of CML patients develop intolerance to imatinib either due to point mutation or gene amplification [43]. Activation of Src-kinases, also contribute to resistance in some cases [44]. To reverse the resistance mechanism and to reduce the side effects of drugs, a promising approach is to combine the conventional chemotherapy with applications of nanotechnology [45]. Palama et al. have used microcapsules for encapsulating imatinib drug and achieved increased drug retention and antitumor activity in CML stem cells and also improved the *ex vivo* purging of malignant progenitors from patient autografts [46].

The up-regulated P-glycoprotein that increases the drug-efflux is considered as the key event for establishment of MDR in cancer cells and to counteract this, P-gp blockers and targeted drug deliverers are the major approaches [47], [48]. The targeted nanoparticles have attracted much attention due to their active targeting property. Selective expression of a cell surface antigen on target cells provides an opportunity for the antibody based therapy for both leukemia and solid tumors [47]. C-type lectin molecules are identified as potential receptors expressed on normal myeloid and leukemic blast cells [36]. Our results also demonstrated about five fold increase in the uptake efficiency after conjugation of lectin glycoprotein to the nanocarriers. This higher uptake of lec-pac-MNPs helped in delivering appropriate therapeutic concentration of paclitaxel to the K562 cells and in turn induced the cytotoxicity effect leading to apoptosis.

Another important parameter in chemotherapy is the availability of drug in the blood circulation and eventually to the target tissues. Studies have shown that many anticancerous drugs have short half-life in body circulation which in turn cripples their potentiality as a drug against cancer [49]. In this regard, nanoparticulate formulations maintain the therapeutic potential of the drug by increasing its bioavailability in serum. In our study, we have compared the kinetics of native pac, pac-MNPs and lec-pac-MNPs and found that the pac-MNPs formulations have a prolonged period of circulation even up to 48 h. Furthermore, after 48 h of treatment, the pac-MNPs treated rats have a therapeutic concentration of paclitaxel in serum whereas the native pac treated rats did not show a detectable concentration. This prolonged bioavailability of nanoformulations is may be due to the fact that pac-MNPs formulations are able to escape the RES system which the native pac cannot do. This suggests that the above pac-MNPs due to its high bioavailability for longer time in serum, helps to enhance the therapeutic index of paclitaxel.

Paclitaxel disrupts the formation of normal spindles at metaphase, leading to arrest of cells at G_2_-M phase of the cell cycle. Jordan et al. have demonstrated that mitotic block induced by low concentrations (10 nM) of paclitaxel results in abnormal mitotic exit and apoptotic cell death [50]. Our results demonstrated higher G_2_-M phase arrest in K562 cells at 10 ng/ml of paclitaxel and the targeted nanoformulation showed higher amount of G_2_-M arrest. The cytotoxicity studies of paclitaxel on K562 cells revealed that pac-MNPs showed lower IC_50_ value than native pac suggesting the efficacy of the nanocarrier system and furthermore, the targeted nanoparticles showed the lowest IC_50_ value suggesting the expediency of targeted drug delivery system (Figure 5). Also, the apoptosis study results obtained from morphological analysis and FACS analysis showed that after 48 h of treatment, the apoptotic population was highest in case of conjugated nanoparticles than that of the unconjugated MNPs and the native drug (Figure 7 A, B).

Our data provides the evidence that paclitaxel could activate both the extrinsic and intrinsic pathways of apoptosis in K562 cells (Figure 11). The extrinsic pathway is not induced by the Fas ligand mediated caspase 8 activation pathway. It has been previously established that caspase-8 is the most proximally activated caspase within the TRAIL mediated death signaling pathway [4] Also, it has been suggested that TRAIL-induced loss of mitochondrial membrane potential was caused by cleavage of Bid via activation of caspase-8 [51]. Bid possesses the biochemical activity to induce cytochrome *c* release by translocating the Bax protein to the membrane of mitochondtria [52]. So, the extrinsic apoptotic pathway might have activated by the TRAIL induced activation of caspase 8. Caspase-8 is thought to propagate the death signal by direct proteolytic processing of downstream caspases such as caspase-3. The activation of caspase 8 dependent apoptotic pathway in Bcr-Abl positive cell line was also confirmed in Kcl22 cells (Figure 10 C). Thus, the extrinsic apoptotic pathway in Bcr-abl positive cell lines involve the caspase 8 dependent cascade. In case of Bcr-Abl negative Jurkat cells also, paclitaxel induces apoptosis through caspase 8 dependent pathway (Figure 10 D). Caspase 8 dependent apoptosis in Jurkat cells after treatment with paclitaxel was also observed by Janssen et al. [53].

The pac-MNPs formulations showed a decrease in the level of Bcr-Abl expression. But, it is known that paclitaxel ascertains its function by phosphorylation of different signaling proteins and Bcr-Abl is a tyrosine kinase which needs to be dephosphorylated for its downregulation. So, paclitaxel cannot directly downregulate the Bcr-Abl. Rather, the experimental results revealed that paclitaxel establishes its antileukemic effects by inducing p38MAPK and JNK pathways in K562 cells. p38MAPK is the mammalian orthologue of yeast HOG kinase that participates in multiple signaling cascades regulating cellular responses generated against cytokines and/or stress-signals [54]. Dumka et al. have demonstrated the key role of p38 kinase for the antileukemic effects of dasatinib [44]. Other studies have also implicated p38 in the multilineage differentiation of Bcr-Abl expressing K562 cells in response to various agents suggesting a key role for p38MAPK in the induction of antileukemic responses in Bcr-Abl transformed cells [55], [56]. The MAPkinases also help in initiating the stress activated JNK pathway. Our results have suggested that with the treatment of paclitaxel nanoformulations, with increasing time period, the level of c-Jun has decreased and the level of p-c-jun has increased (Figure 9 A). Different experimental evidences also suggest that paclitaxel helps in activating the JNK/SAPK signal transduction pathway in various human cell types and induces apoptosis [57]. The activity of JNK is tightly regulated by protein kinases and JNK-specific phosphatases. Phosphorylation of serines 63 and 73 in the c-Jun transactivation domain is known to increase c-Jun activity [58].

Under normal growth conditions, the c-Abl kinase undergoes nucleocytoplasmic shuttling, which may be regulated in part by 14-3-3 binding to the Abl in the cytoplasm (Figure 11). In contrast, the oncogenic Bcr-Abl kinase localizes exclusively in the cytoplasm. C-Jun amino-terminal kinase is known to induce phosphorylation of 14-3-3 proteins disrupting a c-Abl/14-3-3 cytoplasmic complex that leads to nuclear translocation of the oncogenic Bcr-Abl, that leads to genomic instability of *Bcr-Abl* (Figure 11) [38]. The downregulation of Bcr-Abl gene after treatment with pac-MNPs was observed in both the CML cell lines i.e, K562 and Kcl22 (Figure 10 B, C). Inhibition of Bcr-Abl, leads to abrogation of survival pathways, such as Erk1/2, Stat5 and Akt etc. [1]. The downregulation of survival pathways and activation of JNK and p38MAPK stress activated pathways triggers the apoptotic signal cascade (Figure 11).

It is quite evident that lec-pac-MNPs could be used as feasible, safe and effective agents to target CML. The discrete morphology and size as revealed from TEM and by particle size analyzer demonstrated the high stability of the nanocarrier, that could escape from the RES [23], [59]. Further, MNPs have the potential to revolutionize current approaches to clinical diagnosis and therapeutic treatment. MNPs have remarkable potentiality for precise delivery of chemotherapeutic drugs in a targeted manner as these particles can be steered by an external magnetic field to reach the targeted site. Also, these can be used as contrast agent for MRI due to the inherent superparamagnetic property which is helpful in disease detection, monitoring of drug delivery at diseased sites and tumor regression [60]. Previous studies have already proved MRI to be an efficient tool for detection and monitoring of multiple myeloma including leukemia before and after treatment [61], [62]. Ito et al. have shown that early detection of leukoencephalopathy of acute lymphocytic leukemia is possible with the aid of MRI [30]. Our pac-MNPs could also act as image contrast agent in MRI by shortening the T_2_ relaxation time (Figure 2 A, B). With increasing concentrations, the MR intensity gradually decreased. Thus, pac-MNPs could act both as drug delivering and imaging contrast agent. This exemplifies the effectiveness of pac-MNPs as a therapeutic and simultaneously a diagnostic and monitoring tool in the treatment of leukemia.

In conclusion, our study demonstrates for the first time that lectin conjugated paclitaxel loaded MNPs can act on CML cells effectively even at nanogram concentration by persuading higher uptake and targeted delivery of therapeutic concentration. In *in vivo* system the pac-MNPs showed prolonged blood circulation time suggesting the effectiveness of the formulation in evading the RES system and increasing the bioavailability of paclitaxel. Paclitaxel formulations can augment the antileukemic activity by coordinating different apoptotic signaling cascades. The extrinsic apoptotic pathway gets activated in a Fas-L indepdent manner by TRAIL induced activation of caspase 8. Further, the Bcr-Abl oncoprotein, which is causatively linked to CML pathogenesis, becomes instable due to activation of JNK pathway. Besides the therapeutic efficacy, the magnetic nanocarriers could help in early diagnosis and monitoring of multiple myeloma including leukemia before and after treatment. Thus, paclitaxel in combination with magnetic drug carrier can act as a novel theranostic mediator in treatment of chronic myeloid leukemia.

## Materials and Methods

### Materials

Iron (III) chloride hexahydrate (FeCl_3_.6H_2_O), Iron (II) chloride tetrahydrate (FeCl_2_.4H_2_O), 6-coumarin, MTT (3- (4, 5-dimethylthiazol-2-yl)-2, 5-diphenyl tetrasodium bromide) reagent, lectin, DAPI (4', 6-diamidino-2-phenylindole), JC-1 dye and the Annexin-V FITC apoptosis kit were purchased from Sigma-Aldrich (St. Louis, MO). GMO was procured from Eastman (Memphis, TN). N-(3-Dimethylaminopropyl)-N’-ethyl-Carbodiimide hydrochloride (EDC) and N-Hydroxy Succinimide (NHS) were procured from Fluka, Sigma Aldrich, Belgium. Paclitaxel was obtained from Shaanxi Schiphar Biotech Pvt Ltd, China. Primary antibodies for Bcr-Abl, p53, p21, Fas-L, p38kinase and β actin and the secondary antibodies were purchased from Santacruz Biotechnology, CA, USA. The primary antibodies for Caspase 3, Caspase 8, Caspase 9, PARP-1, Bid, Bax, Bad and Bcl-2 were purchased from Cell Signaling, MA, USA. All other chemicals used were of analytical grade obtained from Sigma (St Louis, MO, USA). Milli Q water purged with nitrogen (N_2_) gas was used in all steps involved in the synthesis and formulation of MNPs [15].

### Synthesis and physical characterization of Pac-MNPs

Pac-MNPs were synthesized following our previous protocol [15]. To determine cellular uptake of the pac-MNPs, 6-coumarin was taken as a model fluorescent probe as its efficiency has been already proved [17], [59]. To formulate 6-coumarin-MNPs, 50 µl of 6-coumarin solution (1 mg/ml) was added to the MNP dispersion instead of drugs during formulation. The different physiochemical characterizations like size, physical state of pac inside pac-MNPs were done using TEM and Differential scanning calorimetry (DSC) [15], [27].

The surface properties of the pac-MNPs were observed by an atomic force microscope (JPK nanowizard II, JPK instrument, Bouchestrasse, Berlin, Germany) consisting of pyramidal cantilevers with silicon probes having force constants of 0.2 N/m [63]. Briefly, a sample of nanoparticles (1 mg/ml) was dissolved in PBS (0.1 M, pH 7.4), sonicated (VC 505, Vibracell Sonics, Newton, USA) for 1 minute in an ice bath and then placed on freshly cleaved mica plates. The sample was allowed to dry for 1 hour at room temperature and then scanned using a contact mode cantilever set at frequency 13 kHz and scanned at a speed of 1 Hz. The images were analyzed using JPK data processing software.

### Lectin Conjugation to pac-MNPs

The lectin glycoproteins were conjugated to pac-MNPs and 6-coumarin-MNPs using EDC/NHS chemical conjugation following our previous protocol [15]. The percentage of conjugation was determined using Micro BCA kit (Pierce, Rockford, IL) using a microplate reader (Synergy HT, BioTek Instruments, Inc., Winooski, VT) in a indirect method by measuring the amount of lectin present in the supernatant [59].

### Quantification of drug entrapped and release kinetics study

Quantification of the drug incorporated in the MNPs and its release from the MNPs before and after lectin conjugation was carried out through RP-HPLC following our previous protocol [15]. In brief, ∼1 mg of lyophilized paclitaxel loaded MNPs were dissolved in 1 ml of acetonitrile and sonicated in an ice bath for 1 min, at 55 watt (VC505, Sonics Vibracell, Sonics and Materials Inc., Newtown, USA) and kept in shaker at 37°C at 150 rpm (Wadegati Labequip, India) for 24 h for the entrapped drug to come out from the formulation. After that, the samples were removed and centrifuged for 10 min at 13, 800 rpm at 10°C (Sigma microcentrifuge, 1–15PK, Osterode, Germany). The analysis of samples were done by reverse phase isocratic mode of HPLC with using Agilent 1100 (Agilent technologies, Waldbronn Analytical Division, Germany) which consists of a column (Zorbax Eclipse XDB-C18, 150×4.6 mm, i.d). For estimation of paclitaxel, 20 µl of the sample was injected manually in the injection port and were analyzed with the mobile phase of acetonitrile: water (80∶20 v/v), delivered at flow rate of 1 ml/min with a quaternary pump (Model No -G1311A) at 25°C with thermostat (Model No - G1316A). The drug level was quantified by UV detection at 228 nm with a diode array detector (DAD, Model -G 1315A). The standard curve of paclitaxel was prepared under identical conditions. The amount of paclitaxel in pac-MNPs was determined from the peak area correlated with the standard curve. The release kinetics of paclitaxel was carried out for a time period of three weeks.

### Preparation phantom agar gels for imaging

Phantom agar gels of pac-MNPs were prepared to check the ability of pac-MNPs as contrast agents following our previous protocol and relaxation measurements in phantom gels were done [24]. In brief, different concentration range of pac-MNPs (0–50 µg/ ml) were prepared in PBS (0.1 M, pH = 7.4). A 2.5% w/v agar solution was prepared by heating 500 mg of agar in 20 ml PBS at 80°C for 10 min. For preparing phantom gels, 320 µl of the agar solution was mixed with 1680 µl of pac-MNPs suspension at each concentration and was preheated to avoid gelation while mixing.

### Relaxation measurements of magnetic nanoparticles in phantom gels

To test the T_2_ relaxivity of pac-MNPs different concentrations of pac-MNPs were taken in gel. Further, to test the diagnostic efficiency of pac-MNPs and lec-pac-MNPs in leukemic cells, different concentrations of pac-MNPs and lec-pac-MNPs treated K562 cells were taken in gel for T_2_ relaxation analysis. Tubes containing the gel were positioned in a Bruker Biospec 4.7 T MRI Scanner (BIOSPEC Bruker BioSpin MRI GmbH, Germany) using a 72 mm resonator as transmitter/receiver coil following our previous protocol [24]. To estimate the transverse relaxation time (T_2_) for each sample, coronal images (slice thickness = 2 mm) were acquired at various echo times (TE) from 20 ms to 320 ms with a repetition time (TR) of 10,000 ms. Similarly, T_1_ relaxation time for each sample was measured by varying TR between 15.4 ms and 10,000 ms while keeping TE constant at 10 ms. MR signal intensity were calculated by drawing uniform circular ROIs 0.26 cm^2^).

### Cellular uptake studies

Cellular uptake efficiency was determined by flow cytometry [64]. Briefly, 6 well plates (Corning, NY, USA) were seeded with K562, Kcl22 (both are Bcr-Abl positive) and Jurkat cells (Bcr-Abl negative) at 1×10^5^ cells per well density and incubated at 37°C overnight. The cells were treated with 1 ml of freshly prepared medium containing native 6-coumarin, 6-coumarin-MNPs or lec-6-coumarin-MNPs equivalent to 30 ng/ml of native 6-coumarin and incubated for 2 h at 37°C in CO_2_ incubator (Hera Cell, Thermo Scientific,Waltham, MA). Cells treated with only medium were used as respective controls. At the end of the incubation period, the cells were washed three times with cold DPBS (Dulbecco's Phosphate Buffered Saline) and centrifuged at 1000 rpm (SIGMA 3K30, Munich, Germany) to wash out excess 6-coumarin or 6-coumarin-MNPs or lec-6-coumarin-MNPs, which were not taken up by the cells. The cells were then taken for FACS analysis (FACScan flow cytometer, Becton Dickinson). In all FACS analysis, cell debris and free particles were excluded by setting a gate on the plot of side- scattered light (SSC) vs forward-scattered light (FSC). A total of 10,000 gated cells were analyzed. The increase of fluorescence in the cells treated with MNPs relative to that in the untreated control cells was expressed as mean fluorescence increase relative to control. To substantiate the fact of lectin mediated enhanced uptake, a competitive inhibition assay was carried out in K562 cells (lectin positive cell line) for which, varying concentrations of free lectin (0.5 µg – 5 µg) was added in addition to lectin-6-coumarin-MNPs (30 ng/ml) and the uptake study was carried out. In addition, the lectin receptor mediated uptake was observed in lectin negative HEK293 cell line [36].

### *In vivo* Pharmacokinetics

Pharmacokinetic studies were carried out to study the bioavailability of the native pac, pac-MNPs and lec-pac-MNPs. The experiment on animals was performed with the permission of Institutional Animal Ethics Committee of Institute of Life Sciences, Bhubaneswar, India. The *in vivo* pharmacokinetic study was performed after intravenous injection, (dose- 20 mg/kg) to the Wistar rats (male, ∼300 g, six per experiment). Blood samples were withdrawn from the retro-orbital plexus at various times (30 min, 2 h, 6 h, 24 h and 48 h) and centrifuged using SIGMA 1-15K (Germany) to collect the serum at 12,000 rpm for 1 min at 4°C. To 50 µl of serum 450 µl of acetonitrile was added for deproteinisation and the resulting mixture was again centrifuged at 12,000 rpm for 3 min at 4°C. The supernatant was taken for HPLC analysis using our standardized protocol (already mentioned in quantification of drug entrapped in the materials and method section).

### Cytotoxicity study

To find out the cytotoxicity of the native pac, pac-MNPs and lec-pac-MNPs on K562, Kcl22 and Jurkat cells, mitogenic assay was carried out after 48 h of treatment with varying concentrations of native pac, pac-MNPs and lec-pac-MNPs following our previous protocol [15]. Cells were seeded at 3,000 per well in 96 well plate (Corning, NY, USA) and kept in the incubator for overnight. Different concentrations of native pac, pac-MNPs and lec-pac-MNPs (0.1 ng–µg/ml) were added to the cells and incubated for 48 h at 37°C. After the specified incubation time, 10 µl MTT (Sigma) was added, and the plates were incubated for 3 hours at 37°C in a cell culture incubator following which the intracellular formazan crystals were solubilized in dimethyl sulfoxide and the color intensity was measured at 540 nm using a microplate reader (Synergy HT, BioTek Instruments, Inc., Winooski, VT). The antiproliferative effects of different treatments were calculated as a percentage of cell growth with respect to control.

### Cell cycle Analysis

Cell cycle is an important phenomenon that plays a crucial role in developmental pathways, and is frequently deregulated in many cancer diseases. The distribution of DNA in the cell cycle was studied by flow cytometry [59]. In brief, 2×10^5^ cells/ml were grown in 25 cm^2^ culture flasks (Corning, NY, USA) containing 5 ml media and incubated overnight at 37°C. Next day, 5 ml of media with native pac, pac-MNPs and the lec-pac-MNPs (Concentration- 0.1 ng/ml, 1 ng/ml and 10 ng/ml) were added to the flasks and the cells were incubated for 24 h and 48 h in CO_2_ incubator at 37°C. Cells treated with only medium was used as controls. After incubation time period, the cells were collected and were washed twice with DPBS. The collected cells were stained with propidium iodide. The cell cycle distribution of the cells was determined by analyzing 10,000 gated cells using a FACScan flow cytometer with Cell Quest software (FACS Calibur; Becton-Dickinson, San Jose, CA). All experiments were performed in triplicates.

### Annexin V FITC assay

Percentage of apoptosis was determined by using Annexin V FITC and propidium iodide apoptosis kit (Sigma) [65]. In brief, 5×10^5^ K562 cell/ml were grown in 25 cm^2^ culture flasks (Corning, NY, USA) containing 5 ml media and incubated overnight at 37°C. Next day, the media was removed and 5 ml fresh media containing 10 ng/ml of native pac, pac-MNPs and lec-pac-MNPs were added to the flasks and incubated for 24 h and 48 h. After the particular incubation time period, the cells were collected and washed three times with DPBS (0.1 M, pH 7.4). Then 500 µl of 1×binding buffer was added, to that 5 µl of Annexin V-FITC and 10 µl propidium iodide was added and incubated at room temperature in dark for 10 min. Cells without any drug treatment were taken as negative control. Then flow cytometry analysis was done by analyzing 10,000 gated cells using a FACScan flow cytometer and Cell Quest software (FACS Calibur; Becton-Dickinson, CA, USA). All experiments were performed in triplicates.

### Cell death study by confocal microscopy

In brief, 2×10^5^ K562 cells were seeded in 6 well plates and incubated overnight at 37°C. Next day, the cells were treated with native pac, pac-MNPs and lec-pac-MNPs (10 ng/ml) and incubated for 24 h and 48 h. After the period of incubation, the cells were washed two times with DPBS (0.01 M, pH 7.4) and fixed with formaldehyde and stained with DAPI. Then, the cells were washed and imaging was done with confocal laser scanning microscopy (Leica TCS SP5, Leica Microsystems GmbH, Germany) using the 60 X oil immersion lens with argon laser at 488 nm to detect the nuclei [66].

### Cytofluorometric analysis of mitochondrial membrane potential (Ψm)

Both extrinsic and intrinsic pathways of apoptosis leads to decrease in mitochondrial membrane potential which could help in release of cytochrome C. So, to know the effect of different formulation compared to the native drug, mitochondrial membrane depolarization study was done in K562 cell lines by using flow cytometry [26], [41]. Briefly, 5×10^5^ cells were grown in 25 cm^2^ culture flasks (Corning, NY, USA) containing 5 ml media and incubated overnight at 37°C. The cells were exposed to a particular concentration (10 ng/ml) of native pac, pac-MNPs and lec- pac-MNPs and incubated at 37°C for 24 h and 48 h. After required incubation time, the cells were washed three times with DPBS (0.1 M. pH, 7.4). Then, the cells were re-suspended with 1 ml DPBS (0.1 M pH, 7.4) and the cationic dye JC-1 (3 µg/ml) was added and incubated for 10 min at 37°C to allow a potential-dependent accumulation of the dye in mitochondria. The cells were washed with DPBS (0.1 M, pH 7.4) twice and analyzed by using FACScan flow cytometer and Cell Quest software Caliber (Becton-Dickinson, CA). All experiments were performed in triplicates.

### Immunoblotting to reveal apoptotic pathway

To study the pathway by which apoptosis might have occurred, western blot analysis was performed. In brief, 5×10^5^ K562 cells were grown in 25 cm^2^ culture flasks (Corning, NY, USA) containing 5 ml media and incubated overnight at 37°C. Next day, the media was removed and 5 ml of fresh media containing 10 ng/ml of native pac, pac-MNPs and lec-pac-MNPs were added to the flasks and incubated for 24 h and 48 h. After the time of incubation, protein was isolated following the protocol of Acharya et al. [59]. Protein lysates containing 60 µg of protein were separated by 10% SDS-PAGE and transferred to PVDF membrane. Then, the membrane was blocked with 5% blocking solution (nonfat dry milk for unphosphorylated proteins and Bovine Serum Albumin for phosphorylated proteins) and incubated with different primary antibodies (Bcr-Abl, Caspase 3, Caspase 8, Caspase 9, PARP-1, p53, p21, Fas l, p38 Kinase, c-Jun, Bid, Bcl-2, Bax, Bad and β actin in 1∶1000) for 1 h at room temperature. After that, the membrane was washed and treated with HRP conjugated secondary antibody (1∶5000 dilution) for 40 min at room temperature. The immunoblots were visualized using the enhanced chemiluminescence detection kit (ECL; Amersham, Arlington Heights, IL).

### RT-PCR analysis

RNA was extracted from K562, Kcl22 and Jurkat cells using QuiAmp® RNA blood Mini Kit (Quiagen, Valencia, CA) and the RNA (1.5 µg) was reverse transcribed using First strand cDNA synthesis kit (Fermentas International INC, Ontario, Canada). Quantitative real time PCR was carried out using qPCR ‘Mastermix Plus’ for SYBR Green I-dTTP (Urogentec, Belgium) in 25 µl of PCR reaction mixture containing 1 µl cDNA and primer concentration (6 picomol) using Real time PCR (DNA Engine Opticon2, MJ Research Incorporated, BioRad, Philadelphia, USA). The following primer pairs synthesized from Sigma, USA were used: β-actin: forward, 5’- CTGGCACCACACCTTCTACAAT-3' and reverse, 5'- AATGTCACGCACGATTTCCCGC -3', BCR/ABL: Forward: 5'- GGAGCTGCAGATGCTGACCAAC-3' and Reverse: 5'-TCAGACCCTGAGGCTCAAAGT-3', Bcl2: Forward: 5’- GGA TTG TGG CCT TCT TTG AG -3’ and Reverse: 5’ CCA AAC TGA GCA GAG TCT TC 3’, Caspase -3: Forward: 5’-GCTATTGTGAGGCGGTTGT-3’ and Reverse: 5’-TGTTTCCCTGAGGTTTGC-3’, Caspase 8: Forward: 5’-CGGGATCCGCCACCATGGACTTCAGCAGAAATC-3’ and Reverse: 5’-TCCCCCGGGCACCATCAATCAGAAGGG-3’, Bax: Forward: 5’-TTTTGCTTCAGGGTTTCATC-3’ and Reverse: 5’-GACACTCGCTCAGCTTCTTG-3’ and Bid: Forward: 5’-TGGACTGTGAGGTCAACAACG-3’ and Reverse: 5’-GCGTCCATCCCATTTCTGG-3’. The relative gene copy number was calculated by the concentration-CT standard curve method and normalized using the average expression of β-actin [63].

### Cell Culture

The cell culture experiments were carried out in three leukemia cell lines i.e, K562, Kcl22 and Jurkat and one Human Embryonic Kidney (HEK293) cell line (ATCC, Manassas, VA). Cells were grown in RPMI supplemented with 10% fetal bovine serum, 100 µg/ml penicillin G and 100 µg/ml streptomycin (PAN Biotech Gmbh, Aidenbach, Germany) at 37°C in a humidified and 5% CO_2_ atmosphere maintained in an incubator (Hera Cell, Thermo Scientific, Waltham, MA).

### Statistical Analysis

Statistical analyses were performed using a Student's *t* test. The differences were considered significant for *p* values of<0.05 and very significant for p values of<0.005.
